# A Clinical Lecture on the Causes of Paralysis of the Third Nerve

**Published:** 1906-11-17

**Authors:** George Parker

**Affiliations:** Physician Bristol General Hospital, Lecturer on Medical Jurisprudence, University College, Bristol


### A Clinical Lecture

### ON THE CAUSES OF PARALYSIS OF THE THIRD NERVE.

By GEORGE PARKER, M.A., M.D., M.R.C.S.Eng., Physician Bristol General Hospital, Lecturer on Medical Jurisprudence, University College, Bristol.

Paralysis may affect the whole of the third nerve or some of its branches only; or, again, it may affect other ocular nerves as well as the third. Hence the peculiar strabismus, ptosis, and pupillary states seen in typical cases vary with the extent of the paralysis. Perhaps the most common cause is syphilis, but the condition also frequently follows a simple chill or exposure to cold. It may be due, further, to the pressure of intracranial tumours, to hæmorrhage into the crus cerebri, to tubercular meningitis, or to direct injury, such as gunshot wounds. Other cases follow diphtheria or are associated with tabes, while some are congenital. Hysterical paralyses also occur, and there is one curious recurrent form originally described by Charcot and following attacks of migraine.

Thus the condition is not only of interest to the ophthalmic surgeon, but is important in general medicine.

I may illustrate the variety of the cases we have to deal with from an experience of my own.

I had been attending a young woman for severe headache, strabismus, and ptosis, which improved, relapsed, and improved again. I could exclude syphilis and tumours, but the case was clearly not one of the ordinary types. One evening I was surprised to find another sister, K., in the house with similar symptoms. As she said, laughing, to me, "I can see two mothers in the room instead of one." In the small hours of the night I was summoned hastily to K., and found her dead. The heart indeed beat for a few minutes, but respiration had ceased, and all efforts to resuscitate her were unavailing. She had awakened with agonising pain in the head, and the end came with terrible rapidity. Naturally the other sister feared a like fate, and her terror can be imagined. I knew very little of the dead girl's history, as she had been under the care of someone else, and for the time I could do little to reassure her sister. However, the post-mortem showed a ruptured aneurysm at the junction of the middle cerebral and internal carotid arteries, with copious hæmorrhage. When I saw this I recognised that the chance of two healthy sisters suffering at the same time from such a rare condition as aneurysm at the base of the brain was extremely small, apart from any difference in the history of the cases. Still it was difficult at the time completely to exclude the possibility of such a coincidence. I am thankful to say that the surviving sister is now—18 months later—alive and well, except for some weakness in the ocular muscles. Her affection is, I believe, an instance of Charcot's recurrent ophthalmoplegia.

A comparison of the two cases is important. The patient in whom the aneurysm was found was 24 years of age. She had suffered from what appeared to be neuralgic pains in the head, and had some teeth extracted in January. Then a febrile attack with sore throat, but no joint symptoms or cardiac murmur occurred, though she recovered very slowly. Dr. Staples tells me that the temperature was never more than 100°, but a fortnight later she had some vertigo and vomiting, with severe headaches. For two months she remained unwell, and on March 7th a fainting attack took place. On the 28th a violent headache over the right eye with slight vomiting was noted; and on the 29th double vision with drooping of the eyelid, dilatation of the pupil, and impaired movement of the eyeball. When I saw her again at 1 A.M. her limbs were flaccid and the face cyanosed. Neither artificial respiration, strychnine, nor Faradism had any effect, and in a few minutes the heart ceased to beat. Thus we have a history of some septic infection, with symptoms pointing to the transference of a septic embolus to the brain. The local infection led to an aneurysm which, shortly before or after rupture, compressed and paralysed the third nerve. The sudden death was due to the immense hæmorrhage, for the dura mater was distended with semi-fluid blood clot, the convolutions were flattened, and even below the pia mater large quantities of blood were effused, which covered the base and extended over the occipital lobes and cerebellum. The aneurysm was of the size of a hazel nut. I may remark that the middle cerebral is the vessel most often affected by these cerebral aneurysms, and on that vessel and the basilar together more than half the recorded cases have been found. Rare as they are, they are more common in early life than any other aneurysm, and in each decade of life from 10 to 60 they occur with about the same frequency, while other forms become more common in middle life. Sir William Church long ago pointed out their connection with cardiac trouble, and that they occur at the spot where emboli most easily lodge. Still, as Sir Wm. Gowers remarks, they are in some cases due to hereditary syphilis, or primary fibroid degeneration of the arteries, and about 6 per cent. of them to direct injury, such as fracture of the base. In about 50 per cent. death occurs from rupture, either sudden, gradual, or intermitting. I have seen one other instance in a young girl, probably due to a septic clot. After a slight illness she got out of bed one night and fell dead on the floor from rupture of the aneurysm. The third nerve from its situation is easily pressed on by such aneurysms, as well as by other tumours at the base of the skull or by meningeal inflammation.

To turn now to the case of the other sister. She is aged 22, and has been subject to attacks of right-sided migraine for many years, especially at her periods. About February 20th, 1904, she was troubled with severe pain over the left eye, which got worse, and on March 28th she consulted me about it. The eyelid was slightly drooping, and she vomited two or three times. After a few days complete ptosis came on, with paralysis of the superior, inferior, and internal recti. The pupil was partially dilated. The pain got worse, and resisted all remedies for about a month. Large doses of iodide had no effect, but mercurial inunction over the forehead was followed by sudden cessation of the pain, and a slow recovery from the paralysis. This is the treatment recommended by Charcot in these cases, and I may say that there is no reason to suspect syphilis in this patient. At the end of May, when convalescent at the seaside, an epileptiform convulsion occurred, with unconsciousness for 15 minutes. She was drowsy afterwards, and for some days vomited at intervals. There was no paresis of the limbs, but the headache and ocular paralysis returned. Vomiting was so severe that rectal feeding was employed, the headache was violent, the tongue coated, and the temperature occasionally rose to 100°. Mercurial inunction was again followed by general improvement and decrease of the ocular paralysis. There was nothing abnormal in the optic discs, no history or symptom of mastoid disease, no albuminuria, nothing pointing to syphilis, antrum disease, tuberculosis, or cerebral abscess.

She is now in good health, but has had a few slight attacks of pain over the left eye and sometimes her old migraine over the right. The left pupil is still larger than the other, and the superior rectus has only partially recovered, so that double vision still occurs when she looks upwards. There is no increased dilatation when the neck is stroked, and neither pupil reacts easily to the light. In other respects she is very well. There has never been any paralysis of the limbs, the knee jerks are equal on both sides, but the plantar and toe reflexes in the left foot cannot be obtained. The pulse tension is fairly normal, and there is no cardiac murmur.

This, then, appears to be an instance of Charcot's recurrent type, where the attacks of ptosis come on at intervals with migraine. Swanzy says that the same nerve left or right is affected each time, and that generally all the muscles supplied by it are paralysed. The latter statement is denied by some writers.

Spiller and Posey [1] have recently reported a case of this type which had been preceded by many attacks of migraine, with dimness of vision and nausea. Möbius, who has collected the recent cases, notices that these patients are generally young, and that the attacks may occur several times in a year or at several years' interval. The paralysis may last for weeks or months, and some traces of it may remain permanently. The cause is very uncertain. Charcot and Oppenheim attribute it to vasomotor disturbance and spasm of the blood-vessels. It is rarely fatal. In a few instances, when post-mortems have been made, varied lesions have been found, such as basal pachymeningitis, tubercle, and fibrochondroma. It may be said, however, that such conditions could not cause a chronic affection recurring at intervals. Possibly any local irritation in a person subject to migraine may lead to occasional vasomotor spasm, just as attacks of angina pectoris in anginal patients are brought on by gastric or other irritation. The starting-point is the migrainous diathesis, which is essentially chronic and compatible with long life.

I have dwelt at length on these two rare causes of third-nerve paralysis, and we may now consider briefly what others exist. Congenital and hysterical ptosis without any affection of the other branches of the nerve must not be discussed here, though they involve interesting questions as to treatment.

Tertiary syphilis is a common source of this paralysis, either by producing degeneration of the nuclei, or by the pressure from a gumma or from a syphilitic aneurysm or meningitis, or by direct inflammation of the nerves. The history and the rapidity with which the affection disappears under specific treatment may be our only means of diagnosing this type. I have met with one instance of a very rare variety, the alternating syphilitic form, in a woman whose face was terribly disfigured by specific scars. The third nerve on one side was first affected, and the eye closed. Under mercury and iodide she recovered, but to my surprise I was called on later to treat her for the same trouble in the other eye. There is apt to be a final condition of more or less complete paralysis of both eyes, and, as in all syphilitic brain troubles, the prognosis is uncertain, however quickly and thoroughly they may clear up under medicine.

Patients again are often found with sudden ptosis and strabismus following exposure to cold. This has been ascribed, like so many things, to rheumatism, but the real cause is unknown. However, recovery is usually rapid and complete. In diphtheria, too, paralysis sometimes affects the eye muscles, most often those of accommodation, but, as W. A. Turner points out, either the third or the sixth or the two nerves on both sides of the head may be attacked. Even if the third nerve alone on one side is affected, there is generally a history of previous illness to guide us, and the prognosis is then favourable.

The cases of hemiplegia with paralysis of the third nerve on the opposite side produced by a lesion of the crus will, as a rule, cause no difficulty in diagnosis, though Gowers refers to very rare instances where the third nerve has been affected alone by a small lesion in the crus.

While paralysis from cortical and subcortical lesions is unknown, both acute and chronic nuclear paralyses are fairly common. They are often bilateral, and when acute are associated with symptoms of apoplexy or of the implication of other nerves. "If the internal muscles of both eyes are affected, or the external muscles without the internal, or if the muscles of both eyes which are associated in function are attacked," then we have reason to infer a nuclear origin. Again, a similar affection may mark an early stage of bulbar paralysis, and in chronic nuclear disease we meet with an alternating ptosis like that of syphilis, affecting first one eye and then the other. More or less temporary paralyses occur in tabes and in general paralysis, and may be among the first symptoms of the disease.

Finally, paralysis of the third nerve, with or without the other nerves of the eye, may be due to wounds and to fracture of the base of the skull, as well as to tumours or anything which may exert pressure upon it. Thus we have a large field for investigation, which is difficult from the inaccessibility of the nerve, but the close situation of other nerves leads in most cases to their affection also, and thus points to the site of the lesion. Our first difficulty is to exclude tumours of the brain, then syphilis and other general diseases must be carefully considered as possible causes. The manifestations of disease in all other organs must be searched for if we are to arrive at an accurate diagnosis, and to decide whether it is an unimportant and temporary affection or a sign of the utmost gravity.

[1] Amer. Jour. Med. Sc., Ap. 1904.

